# Supplementary material for: Illumina complete long read assay yields contiguous bacterial genomes from human gut metagenomes
Source: mSystems. 2025 Jul 23;10(8):e01531-24. doi: 10.1128/msystems.01531-24 (PMC12363240; doi:10.1128/msystems.01531-24)
Supplement: Figure S1 — ICLR library preparation workflow. [file msystems.01531-24-s0001.pdf]

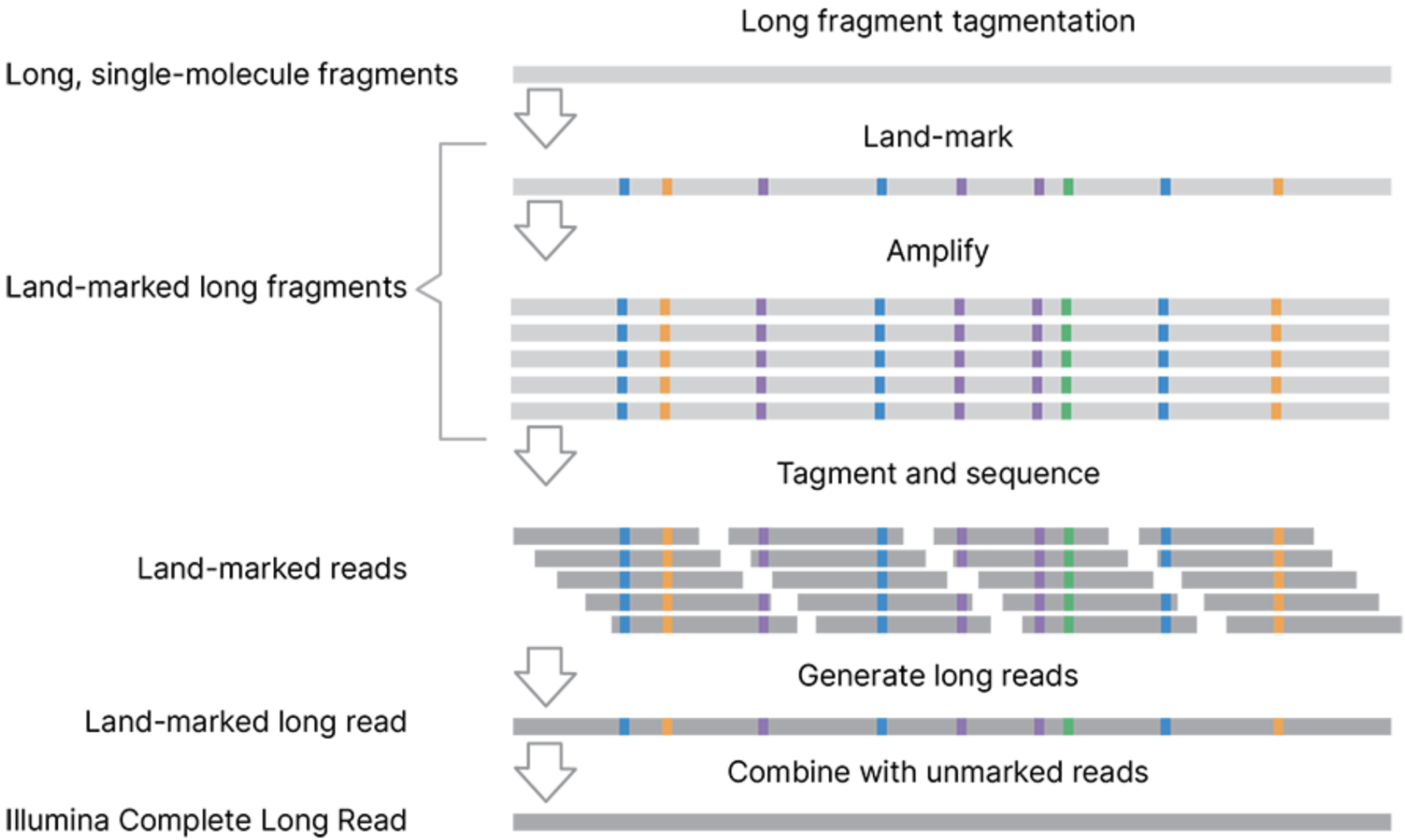

Supplementary Figure 1: ICLR library preparation workflow

Long DNA fragments are marked and amplified. Amplified fragments are tagmented for sequencing. Sequencing reads are reconstructed into long reads using marked bases, and then marks are removed through comparison against unmarked reads. Reproduced with permission from Illumina, Inc.
