# Supplementary material for: Illumina complete long read assay yields contiguous bacterial genomes from human gut metagenomes
Source: mSystems. 2025 Jul 23;10(8):e01531-24. doi: 10.1128/msystems.01531-24 (PMC12363240; doi:10.1128/msystems.01531-24)
Supplement: Figure S3 — Metagenomic assembly graph corresponding to S. cerevisiae genome. [file msystems.01531-24-s0003.pdf]

S.cerevisiae

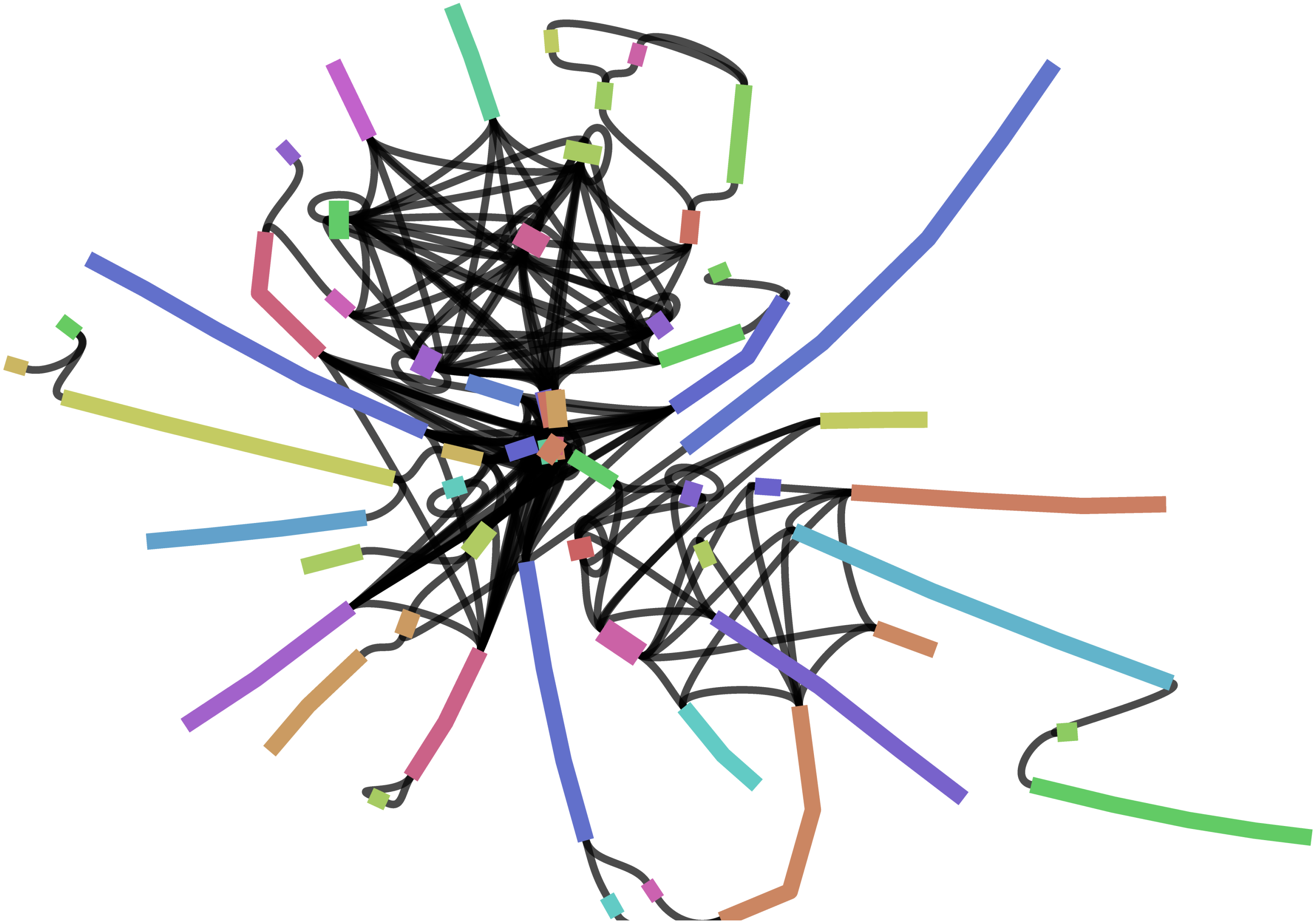

Supplementary Figure 3: Metagenomic assembly graph corresponding to S. cerevisiae genome
The assembly graph of a 10Gb subsampled ICLR was visualized by Bandage.
