# Supplementary material for: Illumina complete long read assay yields contiguous bacterial genomes from human gut metagenomes
Source: mSystems. 2025 Jul 23;10(8):e01531-24. doi: 10.1128/msystems.01531-24 (PMC12363240; doi:10.1128/msystems.01531-24)
Supplement: Figure S4 — Read lengths from human gut metagenomic sequencing. [file msystems.01531-24-s0004.pdf]

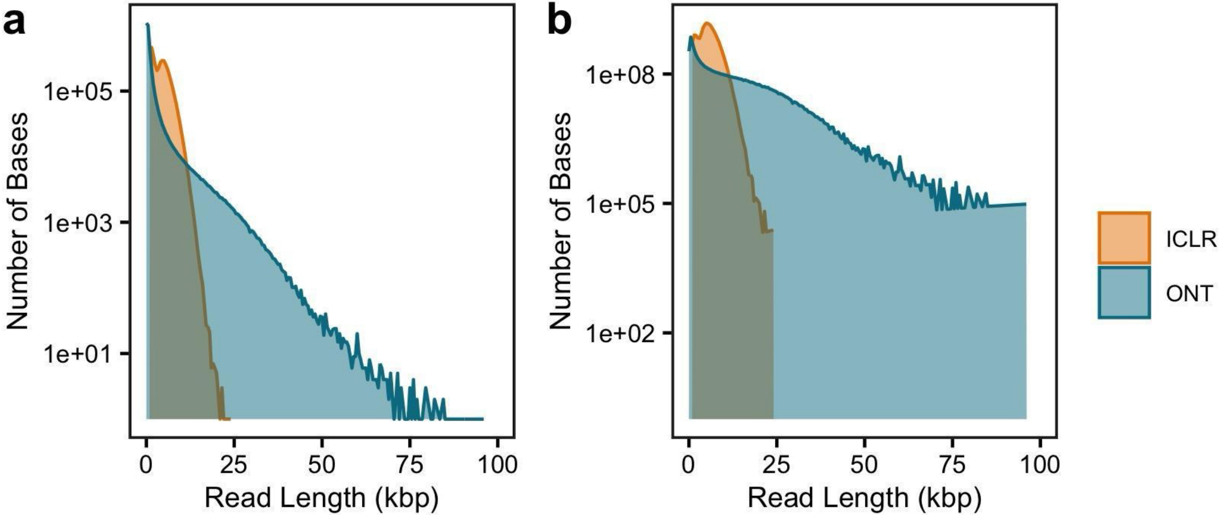

Supplementary Figure 4: Read lengths from human gut metagenomic sequencing
(a) Number of reads of each read length and (b) total bases contained in reads of each length from Illumina Complete Long Read (ICLR) and Oxford Nanopore Technologies (ONT) sequencing of one human gut metagenome (Donor 1). Read and base counts are grouped into bins of five hundred base pairs.
